# Assessment of the choroidal structure in pregnant women in the first trimester

**DOI:** 10.1038/s41598-021-84204-x

**Published:** 2021-02-25

**Authors:** Keiko Azuma, Atsushi Okubo, Takafumi Suzuki, Nozomi Igarashi, Yoko Nomura, Hirotsugu Soga, Hiroshi Murata, Ryosuke Fujino, Asako Ogawa, Haruka Matsui, Takeshi Nagamatsu, Takayuki Iriyama, Ryo Asaoka, Tatsuya Inoue, Maiko Maruyama-Inoue, Kazuaki Kadonosono, Ryo Obata

**Affiliations:** 1 Department of Ophthalmology, Graduate School of Medicine and Faculty of Medicine, The University of Tokyo, Tokyo, 7-3-1, Hongo, Bunkyo-ku, Tokyo, 113-8655 Japan; 2 Department of Ophthalmology and Micro-Technology, Yokohama City University School of Medicine, 4-57 Urafune-cho, Minami-ku, Yokohama, Kanagawa 232-0024 Japan; 3 Department of Obstetrics and Gynecology, Graduate School of Medicine and Faculty of Medicine, The University of Tokyo, Tokyo, 7-3-1, Hongo, Bunkyo-ku, Tokyo, 113-8655 Japan; 4 Department of Ophthalmology, Seirei Hamamatsu General Hospital, Shizuoka, Japan; 5 Seirei Christopher University, Shizuoka, Japan

**Keywords:** Biomarkers, Health care

## Abstract

We investigated the anatomical differences in the choroidal structure between pregnant women in the first trimester of pregnancy and age-matched healthy nonpregnant women using enhanced depth imaging optical coherence tomography (EDI-OCT) and choroidal binarization analysis. The main parameters measured in the two study groups, namely, pregnant women in the first trimester and healthy nonpregnant women, were choroidal thickness and the choroidal luminal area. Binarization of the EDI-OCT images from each patient was performed, and the choroidal vascularity index (CVI) was calculated. The correlations between the baseline characteristics of the subjects and the CVI were investigated using linear mixed model analysis. As a result, there was no statistically significant difference in the mean age, best-corrected visual acuity, axial length, central retinal thickness, subfoveal choroidal thickness, systolic blood pressure (BP), or diastolic BP between the two study groups. Conversely, a significant difference was observed in the CVI (*P* = 0.012) between the two groups. The multivariate analysis identified a significant correlation between the CVI and the systolic BP (*P* = 0.0044, linear mixed test). Taken together, a larger choroidal luminal area was associated with a higher systolic BP, especially in the first trimester of pregnancy. Our findings may provide further insight into the choroidal changes that occur during pregnancy.

## Background

Pregnant women are prone to various retinal and choroidal diseases, because of the dramatic changes in the ocular blood flow that occur during pregnancy^1, 2^, and central serous chorioretinopathy (CSC) is one such disease^3^. Eyes with CSC usually show increased choroidal thickness and increased permeability of the choroidal blood vessels (choroidal vessel hyperpermeability [CVH]), which is a major pathophysiological abnormality underlying the development of CSC^4^. During pregnancy, the choroid might be more susceptible to hemodynamic and hormonal changes because it is a highly vascularized tissue^5^. Therefore, choroidal thickness is often affected during pregnancy. However, the precise changes in the choroid during pregnancy are still unclear, and discrepant results have been reported regarding the ocular blood flow changes that occur during pregnancy^6–10^. Moreover, although hemodynamic and hormonal changes begin to occur during the first trimester of pregnancy, few studies have investigated the changes in the choroidal structure in early pregnancy.

Recently, Sonoda et al. developed a method to binarize optical coherence tomography (OCT) images of the choroid, which enables exclusive investigation of the vessel volume by allowing noninvasive segmentation of the luminal area^11, 12^. This obviates the need for fundus angiography, the use of which is limited in pregnant women because of possible complications^13^. Ever since this technique was introduced, choroidal changes in various macular diseases, such as age-related macular degeneration, have been clarified in detail^14^.

This study aimed to comparatively investigate the choroidal structure in pregnant women in the first trimester of pregnancy and age-matched healthy nonpregnant women using enhanced depth imaging optical coherence tomography (EDI-OCT) with choroidal binarization analyses, which is a noninvasive tool not involving the use of contrast agents. We also attempted to identify if there was any evidence of CVH in early pregnancy.

## Results

Table 1 shows the baseline characteristics of the study subjects. A total of 32 eyes of 16 pregnant women in the first trimester of pregnancy and 26 eyes of 13 age-matched healthy nonpregnant women were included in the current study. None of the pregnant and healthy women showed any evidence of CSC. The mean ages of the pregnant and nonpregnant women were 35.9 ± 4.0 years (mean ± standard deviation; range, 26–43 years) and 35.5 ± 8.7 years (27–52 years), respectively. The mean gestation age in the pregnant women was 11.4 ± 1.2 weeks (9–13 weeks). The spherical equivalents of refractive error were − 0.79 ± 1.9 diopter in the pregnant women and − 2.25 ± 2.8 diopter in the nonpregnant women, indicating no significant difference between the two groups (*P* = 0.10, linear mixed model). The mean axial length (AL) was not significantly different (*P* = 0.20, linear mixed model) between the pregnant (24.1 ± 1.5; range, 22 − 27 mm) and the control groups (24.8 ± 1.4 mm; range, 23 − 27 mm). The mean systolic blood pressures (BPs) in the pregnant and nonpregnant women were 116.8 ± 14.8 (range, 93–160) mmHg and 113.2 ± 19.6 (range, 95–163) mmHg, respectively, and the mean diastolic BPs were 75.5 ± 12.4 (range, 61–110) mmHg and 75.1 ± 10.8 (range, 60–89) mmHg, respectively. There were no significant differences in either the systolic or the diastolic BP between the pregnant and nonpregnant women (*P* = 0.57 and *P* = 0.52, respectively; linear mixed model). The median systolic BPs were 117 (interquartile range [IQR], 112 − 119) mmHg in the pregnant women and 110 (IQR, 100 − 124) mmHg in the nonpregnant women. In the same way, the median diastolic BPs were 74 (IQR 66–80) mmHg in the pregnant women and 71 (IQR 64–81) mmHg in the nonpregnant women.

**Table 1:** Baseline characteristics of the pregnant women in the first trimester of pregnancy and healthy nonpregnant women.

Variables	Total	Pregnant	Control	*P* value
No. of eyes	58	32	26	
Age, years	35.7 ± 6.5	35.9 ± 4.0	35.5 ± 8.7	0.88
AL, mm	24.4 ± 1.5	24.1 ± 1.5	24.8 ± 1.4	0.20
LogMAR VA	− 0.11 ± 0.05	− 0.11 ± 0.06	− 0.09 ± 0.03	0.33
Systolic BP, mmHg	115.1 ± 17.1	116.8 ± 14.8	113.2 ± 20.0	0.57
Diastolic BP, mmHg	74.4 ± 11.7	75.5 ± 12.4	73.2 ± 10.8	0.52

Data are presented as mean ± standard deviation.

AL, axial length; BP, blood pressure; LogMAR, logarithm of the minimal angle of resolution.

The mean central retinal thicknesses (CRTs) were 258.7 ± 11.3 μm in the pregnant women and 267.3 ± 16.8 μm in the nonpregnant women, with no statistically significant difference of the CRT between the two groups (*P* = 0.11, linear mixed model). The mean subfoveal choroidal thicknesses (SFCTs) were 311.1 ± 88.5 μm in the pregnant women and 291.9 ± 82.2 μm in the nonpregnant women, with no significant difference between the two groups (*P* = 0.54, linear mixed model). The mean choroidal vascularity index (CVI) values in the pregnant and nonpregnant women were 63.9% ± 3.8% and 60.8% ± 1.7%, respectively, and the univariate analysis suggested that there was a statistically significant difference between the two groups (*P* = 0.012, linear mixed model) (Table 2). In the multivariate analysis performed with eight explanatory variables (age, subject type [pregnant/nonpregnant women], AL, logMAR VA, CRT, SFCT, systolic BP, and diastolic BP), the subject type (pregnant/nonpregnant women) and systolic BP were found to be significantly associated with the CVI (*P* = 0.0045, and *P* = 0.0044, respectively; linear mixed model) (Table 3). More specifically, a larger CVI was associated with a higher systolic BP and pregnancy.

**Table 2:** Anatomical baseline values in the pregnant women in the first trimester and the control age-matched healthy nonpregnant women.

Variable	Total	Pregnant	Control	*P* value
CRT, µm	262.6 ± 14.6	258.7 ± 11.3	267.3 ± 16.8	0.11
SFCT, µm	302.5 ± 85.5	311.1 ± 88.5	291.9 ± 82.2	0.54
CVI, %	62.5 ± 3.4	63.9 ± 3.8	60.8 ± 1.7	0.012

Data are presented as mean ± standard deviation.

CRT, central retinal thickness; SFCT, subfoveal choroidal thickness.

**Table 3:** AICc model selection for the choroidal CVI.

Parameter	Coefficient	SE	*P* value
Age, years	NS		
Pregnant or the control	2.86	3.1	0.0045
AL, mm	NS		
LogMAR VA	NS		
CRT, µm	NS		
SFCT, µm	NS		
Systolic BP, mmHg	0.14	3.1	0.0044
Diastolic BP, mmHg	NS		

SE, standard error; AL, axial length; LogMAR, logarithm of the minimal angle of resolution; VA, visual acuity; CRT, central retinal thickness; SFCT, subfoveal choroidal thickness; BP, blood pressure; NS, not selected in the optimal model.

Then, we separately analyzed the factors associated with the CVI in the pregnant and nonpregnant women. As shown in Table 4, in the pregnant women, only systolic BP was selected in the optical model for the CVI (*P* = 0.0013). Conversely, none of these variables was selected in the optimal model for the CVI in the nonpregnant women. Thus, the correlation between systolic BP and CVI in the pregnant women persisted even after adjustments for the baseline parameters.

**Table 4:** AICc model selection for the CVI in pregnant women in the first trimester of pregnancy and healthy age-matched nonpregnant women.

Variable	CVI in pregnant women			CVI in control women		
Parameter	Coefficient	SE	*P* value	Coefficient	SE	*P* value
Age, years	NS			NS		
AL, mm	NS			NS		
LogMAR VA	NS			NS		
CRT, µm	NS			NS		
SFCT , µm	NS			NS		
Systolic BP, mmHg	0.28	0.07	0.0013	NS		
Diastolic BP, mmHg	NS			NS		

SE, standard error; AL, axial length; LogMAR, logarithm of the minimal angle of resolution; VA, visual acuity; CRT, central retinal thickness; SFCT, subfoveal choroidal thickness; BP, blood pressure; NS, not selected in the optimal model.

The multivariate analysis identified the AL as being significantly associated with the SFCT (*P* = 0.0022, linear mixed model), but not with any of the other variables of age, subject type (pregnant/nonpregnant women), CRT, SFCT, systolic BP, and diastolic BP (Table 5).

**Table 5:** AICc model selection for the SFCT.

Parameter	Coefficient	SE	*P* value
Age, years	NS		
Pregnant or the control	NS		
AL, mm	− 41.0	− 3.4	0.0022
LogMAR VA	NS		
CRT, µm	NS		
CVI, %	NS		
Systolic BP, mmHg	NS		
Diastolic BP, mmHg	NS		

SE, standard error; AL, axial length; BP, blood pressure; CRT, central retinal thickness; LogMAR, logarithm of the minimal angle of resolution; NS, not selected in the optimal model; VA, visual acuity.

## Discussion

In the present study, we compared the choroidal structure between pregnant women in the first trimester and age-matched nonpregnant women. The results of our analysis revealed a significant difference in the CVI but not in the SFCT between the pregnant and nonpregnant women. Factors associated with the CVI were separately analyzed in the two groups, which revealed a significant correlation between systolic BP and CVI in the pregnant women. It is important to understand the changes in the choroidal structure during pregnancy because pregnant women are more susceptible to retinal and choroidal diseases, such as CSC.

Previous studies that have investigated SFCT in pregnancy have reported conflicting results. Takahashi and Benfica reported that there was no significant difference in the SFCT between women in the third trimester of pregnancy and healthy nonpregnant women^7, 15^. By contrast, Rothwell et al. suggested that the SFCT was significantly higher in pregnant women in the third trimester compared to that in age-matched healthy nonpregnant women^16^. Goktas et al. reported that the SFCT was significantly lower in pregnant women in the second trimester compared to that in nonpregnant women, whereas no such difference was observed between women in the first and second trimesters of pregnancy^17^. By contrast, in the current study, no significant difference in the SFCT was observed between pregnant women in the first trimester and nonpregnant women. In contrast to the SFCT, there was a significant difference in the choroidal CVI between pregnant women in the first trimester and nonpregnant women in the current study, suggesting that the change in the ocular blood flow may more sensitively reflect the CVI than the SFCT. Moreover, an increase in the choroidal CVI might precede the increase in the SFCT during pregnancy. Various hemodynamic changes occur during pregnancy, in addition to hormonal, metabolic, and cardiovascular changes^10^. Of note, the vascular resistance decreased with the establishment of pregnancy, because of hormonal changes^8, 18^, which results in the highest ratios of the blood flow to the tissue volume in the body, including that in the choroidal circulation^5^. Su et al.^19^ suggested that there was no significant difference in CVI in uncomplicated pregnant women compared with that of age-matched normal controls. However, they examined the pregnant women in late pregnancy, whereas the current study enrolled the participants during early pregnancy. The current results are partially in agreement with these previous studies, and it would be of interest to investigate the changes in the SFCT and CVI in the later stages of pregnancy in a future study, as it was beyond the scope of the current study.

In the current study, we found no significant correlation between the SFCT and systolic BP in the first trimester of pregnancy. However, there was a significant correlation between the CVI and systolic BP in the first trimester of pregnancy. There has been no previous investigation on the association between the CVI and systolic BP during pregnancy. The relationship between the SFCT and systolic BP in healthy subjects remains controversial^20, 21^. Li et al. reported that there was no significant correlation between the systolic BP and SFCT^22^. Polak et al. found a significant increase in the choroidal blood flow with increasing BP using color Doppler imaging^23^. The current study suggested that the influence of systolic BP on the choroidal blood flow was reflected sensitively on the CVI, but not on the SFCT, in pregnant women in the first trimester.

**Figure 1:**
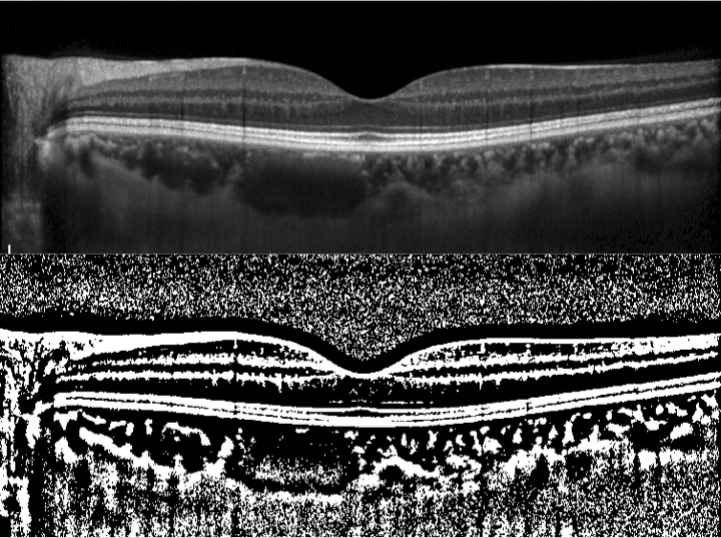
OCT and binarized images of a 38-year-old pregnant woman in the 12th week of pregnancy. In the third trimester, she was diagnosed with preeclampsia. (Top) EDI-OCT image of the horizontal line scan through the fovea in the 12th week of pregnancy. The SFCT was 281 µm and the CVI was 70.4%. (Bottom) Binarized image of the EDI-OCT image.

Preeclampsia is a pregnancy complication characterized by high BP and potential damage of various organs systems, such as the liver and kidney, and dysfunction of the choroidal circulation has also been reported. Some previous studies have compared the SFCT in healthy pregnant women, pregnant women with preeclampsia, and healthy nonpregnant women^24–26^. Most studies have suggested that the choroidal thickness in healthy pregnant women is higher compared to that in healthy nonpregnant women, whereas that in pregnant women with preeclampsia is lower. By contrast, one study by Kim et al. suggested that the choroidal thickness in pregnant women with preeclampsia was higher compared with that in healthy pregnant and healthy nonpregnant women^25^. In the current study, patients with preeclampsia were enrolled, as shown in Fig. 1. The inclusion of preeclampsia cases might have had an influence on our results with respect to the SFCT and CVI to some extent.

Our study has several limitations. First, the sample size was relatively small. Second, the follow-up period was limited to the first trimester of pregnancy. Further analysis of the CVI, in addition to the SFCT, in a future study with a larger sample size and longer follow-up period would be needed to clarify the structural changes in the choroid during pregnancy.

## Conclusions

In pregnant women in the first-trimester of pregnancy, the CVI was significantly increased as compared to that in age-matched healthy nonpregnant women. Moreover, the increased CVI was associated with the elevation of the systolic BP in early pregnant women. Considering the fact that pregnant women have increased risk of CSC, the association between CVI and systolic BP might be a clue to resolve the pathophysiology of CSC.

## Methods

### Participants and enrollment criteria

This prospective study was conducted with the approval of the Institutional Review Board of the University of Tokyo. All data were fully anonymized before we accessed them. We confirmed that all research was conducted in accordance with relevant guidelines or regulations, and informed consent was obtained from all participants. Our study was conducted in accordance with the Declaration of Helsinki. Participants who did not grant authorization for the use of their medical records in the research were excluded from the analyses. We reviewed the medical histories of the women in the first trimester of pregnancy and healthy nonpregnant women at the outpatient clinic of the University of Tokyo Hospital. The inclusion criteria for the pregnant women were as follows: (1) 9 to 13 weeks of gestation, (2) eyes with a spherical equivalent between − 6 diopters and + 3 diopters, and (3) eyes with clear ocular media. The exclusion criteria were the presence of other eye diseases (e.g. chorioretinal atrophy in the macula, glaucoma, and any other retinal disorders) and high myopia (≥ − 6.0 diopters). None of the participants had been diagnosed with CSC prior to the study entry.

### Eye examination

Each participant underwent a comprehensive ophthalmological examination, including measurement of the best-corrected visual acuity, AL (IOL Master, Tomey OA-2000, version 5.4.4.0006; Tomey, Nagoya, Japan), and refractive error (KR-8900 version 1.07; Topcon Corp., Tokyo, Japan), and measurement of the CRT and SFCT by OCT (HRA Spectralis; Heidelberg Engineering GmbH, Dossenheim, Germany) using the EDI technique. The EDI-OCT examinations in all cases were performed between 0900 and 1100 h to avoid the confounding effects of the diurnal fluctuations of the choroidal thickness. Each participant’s age and BP were also recorded at the time of the imaging^27, 28^. The current study did not include the measurement of the intraocular pressure, which could have altered the choroidal structure, considering minimal invasion for the pregnant women.

EDI-OCT images of the choroid were obtained at the fovea, and binarization of the images was performed using the modified Niblack method using the ImageJ software, as described in a previous study^12, 29^. In brief, an image of one central scan passing through the fovea was selected and then converted into 8 bits, and the Niblack auto-local threshold was applied to binarize the images and separate the choroidal luminal and stromal areas. The selection and analyses were independently made by two blinded graders (KA and AO). After binarization, the total choroidal area, luminal area, and CVI were calculated.

### Statistical analysis

The age, AL, logMAR VA, systolic BP, and diastolic BP of both the pregnant and age-matched healthy nonpregnant women were analyzed by the likelihood ratio test using a linear mixed model. All statistical analyses were conducted using the statistical programing language “R” (version 3.1.3; The R Foundation for Statistical Computing, Vienna, Austria). The relationships between the CVI and eight explanatory variables (age, subject type [pregnant/nonpregnant woman], AL, logMAR VA, CRT, SFCT, systolic BP, and diastolic BP) were evaluated using univariate and multivariate linear regression models. Similarly, the relationships between the SFCT and eight explanatory variables (age, subject type [pregnant or nonpregnant], AL, logMAR VA, CRT, CVI, systolic BP, and diastolic BP) were also evaluated using a linear regression model. Then, the optimal linear model was selected among all possible combinations of predictors: 2^8^ patterns, based on the second-order bias-corrected Akaike information criterion (AICc) index (denotes optimal model). The AIC is a well-known statistical parameter used for model selection, and the AICc is corrected AIC, which provides an accurate estimation even when the sample size is small^30^. The degrees of freedom in a multivariate regression model decrease as the number of variables increases. Therefore, the use of the model selection method has been recommended to improve the model fit by removing redundant variables^31, 32^.

### Ethics declaration

The study was conducted in accordance with the tenets of the Declaration of Helsinki and with the approval of the ethics committee at the coordinating center of the University of Tokyo.

### Consent to participate

All patients provided written informed consent prior to participation in the study.

## Data Availability

The datasets generated and/or analyzed during this study will be made available by the corresponding author upon reasonable request.

## Abbreviations

AL: Axial length
BP: Blood pressure
LogMAR: Logarithm of the minimal angle of resolution
SD: Standard deviation
CRT: Central retinal thickness
SFCT: Subfoveal choroidal thickness
NS: Not selected in the optimal model
VA: Visual acuity
